# Comparing vitamin D receptor gene polymorphisms in rs11568820, rs7970314, rs4334089 between COVID-19 patients with mild and severe symptoms: a case control study

**DOI:** 10.1038/s41598-024-57424-0

**Published:** 2024-05-03

**Authors:** Noushin Mohammadifard, Ladan Sadeghian, Razieh Hassannejad, Elham Khosravi, Mojgan Gharipour, Simin Karimi, Shidokht Hosseini, Mahtab Sepahifar, Ghazaleh Bahrami, Fahimeh Haghighatdoost, Nizal Sarrafzadegan

**Affiliations:** 1 Isfahan Cardiovascular Research Center, Cardiovascular Research Institute, Isfahan University of Medical Sciences, Isfahan, Iran; 2 Interventional Cardiology Research Center, Cardiovascular Research Institute, Isfahan University of Medical Sciences, Isfahan, Iran; 3 Heart Failure Research Center, Cardiovascular Research Institute, Isfahan University of Medical Sciences, Isfahan, Iran; 4 Student Research Committee, Isfahan University of Medical Sciences, Isfahan, Iran

**Keywords:** Vitamin D receptor, Single nucleotide polymorphisms, SNP, COVID-19, Genetics, Pathogenesis

## Abstract

The associations of vitamin D receptor (VDR)- single nucleotide polymorphisms (SNPs) with the symptoms of COVID-19 may vary between patients with different severities of COVID-19. Therefore, in the present study, we aim to compare VDR polymorphisms in severe and mild COVID-19 patients. In this study, a total number of 85 hospitalized patients and 91 mild/moderate patients with COVID-19 were recruited. SNPs in VDR genes were determined using ARMS and then confirmed by sanger sequencing. The mean (SD) age of participants in hospitalized and non-hospitalized group was 59.0 (12.4) and 47.8 (14.8) years, respectively. Almost 46% of participants in hospitalized and 48% of participant in non-hospitalized group were male. The frequency of TT genotype of SNP rs11568820 was significantly lower in hospitalized than non-hospitalized group (3.5% vs. 17.6%; *P* = 0.018). However, there was no significant differences between genotypes of SNPs rs7970314 and rs4334089 and also alleles frequencies in all SNPs of two groups. The genotype of rs11568820 SNP had an inverse association with hospitalization of patients with COVID-19 after adjustment for comorbidities [OR 0.18, 95% CI 0.04, 0.88; *P* = 0.034]. While, there was no relationship between genotypes of SNPs rs7970314 and rs4334089 and hospitalization. The TT genotype of rs11568820 plays protective role in sever COVID-19 and hospitalization. Further studies with a large sample size which consider various confounding factors are warranted to confirm our results.

## Introduction

The clinical symptoms of COVID-19 vary from none or mild to severe pneumonia or death^1^. Although current evidence suggests no association between serum vitamin D concentration and susceptibility to COVID-19 infection, its deficiency is more frequent among individuals with severe COVID-19 symptoms^2^. The detrimental effects of vitamin D deficiency on viral respiratory tract infections and the favorable effects of vitamin D supplementation on acute lung injury have been shown previously^3–5^. The active form of vitamin D can be produced by lungs owing to the expression of 1α-hydroxylase in the bronchial epithelium and immune cells. Vitamin D activated in lungs exerts immunomodulatory functions via stimulating the secretion of antimicrobial peptide cathelicidin, preventing from dendritic cell and T cells activation and reducing chemokine secretion^3^.

Most of available evidence on vitamin D association and COVID-19 examined its serum levels, while different polymorphisms involved in the pathways of vitamin D metabolism can affect both innate and adaptive immune responses^6^. Vitamin D receptor (VDR) gene polymorphisms are one of the most widely studied polymorphisms in relation to immune function. VDR acts as a transcriptional factor for around 5% of human genes via binding to VDR elements (VDREs). Any variation in single nucleotide polymorphisms (SNPs) in functional region of the gene can potentially affect VDR function, stability and expression. VDR needs to be expressed and activated to bind with its main ligand, that is 1, 25(OH)2D3 to exert its effects. VDRs are present in almost all immune cells, including activated CD4+ and CD8+ T cells, B cells, neutrophils, and antigen-presenting cells (APC) such as macrophages and dendritic cells^7^. The alleles of the *FokI* polymorphism influences vulnerability to virus infection^6^. The presence of the T allele mitigates the ability of the VDR to bind with gene components which is responsible for vitamin D effects and increases vulnerability to virus infection^6^.

In a cross-sectional analysis among 500 COVID-19 patients with different severities, the associations of VDR-SNPs with the symptoms of COVID-19 varied between patients with different severities^8^. For instance, shortness of breath was associated with the VDR-SNPs of *ApaI*, *CDX2* and *Tru9I*, renal disease with *BsmI* and hypertension with *FokI* and *CDX2*, whereas *TaqI* and *BglI* polymorphisms were not related to any of the symptoms^8^. In addition, COVID-19, as a virus of the coronavirus family, leads to respiratory infections. The influence of genetic SNPs on vitamin D pathway results in susceptibility to upper respiratory infections, as well as lower respiratory infections. Some studies have indicated the association of these selected SNPs with risk of respiratory infections^9,10^.

The detection of genes variants in relation to the susceptibility to COVID-19 infection and its outcomes can shed light on the treatment strategies and identifying susceptible individuals for prevention and provide an insight into future research to combat the virus. Therefore, in the present study, we aimed to compare VDR polymorphisms in severe and mild COVID-19 patients.

## Materials and methods

### Design and participants

This study was conducted on patients with COVID-19 who were recruited in the 5 years Isfahan Covid Cohort (ICC) study. Confirmed patients aged 19 and over in Isfahan with a positive real time polymerase chain reaction (RT-PCR) were eligible to be included in the present study. Patients were selected using convenience sampling method among the provincial health center dataset that registered every patient with positive COVID test. The details of study design and sampling were presented previously^11^. In the current study, the power analysis was performed based on the *CDX2* (*rs11568820*) polymorphism^8^. The analysis considered a power level at 85%, α at 0.05 and a clinical difference between two groups of at least 0.2. The adequate sample size was estimated to be approximately equal to 96 patients in each group (hospitalized vs. non-hospitalized) and a total number of 200 patients with COVID-19 were enrolled in the study.

Subjects were invited to healthcare centers of Isfahan or the Isfahan Cardiovascular Research Institute clinics after one-month infected. They were divided into two groups hospitalized and non-hospitalized patients. Patients were hospitalized based on the WHO definition. According to World Health Organization (WHO) criteria, those with a respiratory frequency of > 30 breaths per minute, SpO_2_ < 94% on room air at sea level, a ratio of arterial partial pressure of oxygen to fraction of inspired oxygen (PaO_2_/FiO_2_) < 300 mmHg, or lung infiltrates > 50% were considered moderate or severe cases and hence, hospitalized^12^. Non-hospitalized patients consisted of individuals whose RT-PCR test was positive for SARS-CoV-2 but had no symptoms^12^ or had any of the various signs and symptoms of COVID-19 (e.g., fever, cough, sore throat, malaise, headache, muscle pain) but not shortness of breath, dyspnea, or abnormal chest imaging^12^. A written informed consent form was obtained from all participants before the enrollment in the study. This study was performed in accordance with the declaration of Helsinki. The study protocol was reviewed and approved by the ethic committee of National Institutes for Medical Research Development (NIMAD), Tehran, Iran (IR.NIMAD.REC.1399.282).

### Data collection

In the present study, all data were collected by the interviewers working at health centers who received a full day training session in terms of questionnaires filling and performing measurements.

### Demographic, socioeconomic and lifestyle behaviors questionnaire

Data regarding demographic and socioeconomic status were collected. Using valid and reliable questionnaires, smoking and physical activity were assessed. Tobacco smoking habit was determined using global adult tobacco survey (GATS) questionnaire which was designed by Centers for Disease Control and Prevention (CDC) and WHO^13^. Drug abuse was assessed using a validated questionnaire in Iran^14^ and physical activity level was measured using the international physical activity questionnaires (IPAQ)^15^. Anthropometric measurements, including height, weight, and waist circumference were determined in health centers according to the standard protocols. Body weight was measured using a standard digital scale and recorded to the nearest 0.5 kg. Height was measured using an inelastic tape measure and recorded to the nearest 0.5 cm^16^. Body mass index (BMI) was calculated by dividing weight (Kg) to height (m) square. Waist circumference (WC) was measured at the at the approximate midpoint between the lower margin of the last palpable rib and the top of the iliac crest using an inelastic tape to the nearest 0.1 cm. Using OMRON barometer, the blood pressure was measured by trained nurses while participants were sitting and rested at least for 5 min. Measurements were performed twice and the mean value of two measurements was recorded as the final vale for blood pressure.

### Medical history

All medical history and the existence of NCDs including cardiovascular disease (CVD), hypertension (HTN), diabetes mellitus (DM), chronic respiratory disease (CRD) and chronic kidney disease (CKD), as well as the history of medicines were obtained by interviewing patients in health centers.

### Blood measurement

All blood samples will be transferred to the central laboratory in Isfahan Cardiovascular Research Institute. Using the salting-out approach, DNA was isolated from 500 µl of peripheral blood^17^. Nanodrop and agarose gel were then used to assess the quality of the DNA. In the next step, primers with the following sequences were designed to genotype exons 21 and 26: Forward 5-TCTCATGAAGGTGAGTTTTC-3, and reverse 5-AGAGCATAGTAAGCAGTAGG-3; Forward 5-CCTTAATCTCACAGTAACTTGGCA-3, and reverse 5-AAGGGTGTGATTTGGTTGCT-3, respectively. The PCR materials included 1.6 μL DNA, 20 μL of mastermix (Ampliqon A/S co.), 0.7 l of each primer, and distiled water up to 40 μL with the following instructions: 95 °C for 5 min, 30 cycles of 95 °C for 45 s, 59 °C for 40 s, and 72 °C for 30 s, and 72 °C for 80 s as the final extension. Finally, the PCR products were sequenced bidirectionally by the Sanger technique utilizing Device (Illumina NGS platform) after confirming the quality and quantity on the gel electrophoresis.

### Statistical analysis

Data were expressed as percentage for categorical and mean ± standard deviation (SD) for continuous variables. The differences between hospitalized and non-hospitalized groups were examined either applying a Chi-square or Fisher-exact tests for categorical variables or independent sample t-test for continuous variables. We applied the Mann–Whitney test to evaluate means across groups when the assumption of normality was not met. In addition, we use Bonferroni correction for these χ^2^-tests for comparing distribution. The bivariate analysis of association between the hospitalization and the polymorphisms was performed with multiple models (genotypic, recessive, dominant and allelic) using the Chi-square test. The crude and adjusted odds ratio (OR) and 95% confidence intervals (CIs) for hospitalization across genotypes of polymorphisms were estimated by applying logistic regression. Three logistic regression models were considered for each SNP (genotypic, recessive and dominant). Statistical analyses were performed using Statistical Package for Social Sciences (SPSS) version 25 (IBM Corp, Armonk, NY, USA. Available at: https://www.ibm.com/support/pages/downloading-ibm-spss-statistics-25), and STATA software, version 14 (available at: https://www.stata.com/stata14/). *P* < 0.05 (two-tailed) were considered as statistically significant.

## Results

Totally 176 patients with COVID-19 including 85 hospitalized patients and 91 non-hospitalized patients were recruited for the study. Participant characteristics are summarized in Table 1. The mean age and WC were higher and physical activity level was lower in the hospitalized group than non-hospitalized group (59.04 ± 12.40 vs. 47.77 ± 14.79 years, 101.25 ± 11.58 vs. 98.24 ± 10.40 cm and 15.59 ± 52.16 MET-h/wk vs. 19.92 ± 63.82 MET-h/wk, respectively). Moreover, the frequency of current smoker was lower and hypertension and diabetes mellitus were higher in the hospitalized versus non-hospitalized group (1.2% vs. 11.0, 43.1 vs. 20.0 and 40.3 vs. 11.1, respectively).

**Table 1:** General characteristics of participants based on hospitalized and non-hospitalized patients with COVID-19.

	Hospitalized N = 85	Non-hospitalized N = 91	*P* value
Age (y)	59.04 ± 12.40	47.77 ± 14.79	< 0.001
Male, n (%)	37 (45.7)	44 (48.4)	0.726
Married, n (%)	71(87.7)	75 (82.4)	0.60*
Education status n (%)			0.034
> 12 y	19 (23.5)	35 (38.5)	
≤ 12 y	62 (76.5)	56 (61.5)	
Smoking status n (%)			0.808
None smoker	76 (93.8)	80 (87.9)	0.009*
Ex-smoker	4 (4.9)	1 (1.1)	
Current smoker	1 (1.2)	10 (11.0)	
Obesity status n (%)			0.808
Normal weight	15 (18.8)	20 (22.0)	
Overweight	40 (50.0)	46 (50.5)	
Obese	25 (31.3)	25 (27.5)	
Abdominally obese n (%)	59 (72.8)	58 (63.7)	0.201
BMI (kg/m^2^)	28.51 ± 4.39	27.98 ± 4.40	0.491**
WC (cm)	101.25 ± 11.58	98.24 ± 10.40	0.043**
Physical activity level (MET-h/wk.)	15.59 ± 52.16	19.92 ± 63.82	< 0.001**
Comorbidities n (%)			
Cardiovascular disease	14 (19.4)	9 (10.0)	0.087
Hypertension	31 (43.1)	18 (20.0)	0.002
Diabetes mellitus	29 (40.3)	10 (11.1)	< 0.001
Chronic respiratory disease	2 (2.8)	1 (1.1)	0.585
Chronic kidney disease	14 (19.4)	19 (21.1)	0.794

*BMI* body mass index, *WC* waist circumference.

*Fisher Exact test; **Mann Whitney test.

### Single nucleotide polymorphisms and their association with COVID-19 hospitalization

The comparison of frequency of patients with different genotypes of various studied SNPs are shown in Table 2. The frequency of TT genotype of rs11568820 SNP was significantly lower in hospitalized than non-hospitalized group (3.5% vs. 17.6%; *P* = 0.018). Also, the recessive model showed statistical significance for this SNP (96.2% vs. 82.4%; *P* = 0.003). However, there was no significant differences between genotypes of SNPs rs7970314 and rs4334089 and also alleles frequencies in all SNPs of two groups.

**Table 2:** Genotype and allele frequencies of different single nucleotide polymorphisms of vitamin D receptors in patients with COVID-19 based on hospitalization.

SNPs and allele frequency	Models	Genotype	Non-hospitalized N (%)	Hospitalized N (%)	*P* value
rs11568820	Genotypic	CC	46 (50.5)	43 (50.6)	0.006
		CT	29 (31.9)	39 (45.9)	
		TT	16 (17.6)	3 (3.5)	
	Dominant	T	45 (49.5)	42 (49.4)	0.996
		CC	46 (50.5)	43 (50.6)	
	Recessive	TT	16 (17.6)	3 (3.5)	**0.003**
		C	75 (82.4)	82 (96.2)	
	Allele	C	121 (66.5)	125 (73.5)	0.150
		T	61 (33.5)	45 (26.5)	
rs7970314	Genotypic	AA	36 (39.6)	35 (41.2)	0.175
		AG	35 (38.5)	40 (47.1)	
		GG	20 (22.0)	10 (11.8)	
	Dominant	G	55 (60.4)	50 (58.8)	0.827
		AA	39 (39.6)	35 (41.2)	
	Recessive	GG	20 (22)	10 (11.8)	0.072
		A	71 (78)	75 (88.2)	
	Allele	**A**	107 (58.8)	110 (64.7)	0.254
		**G**	75 (41.2)	60 (35.3)	
rs4334089	Genotypic	CC	48 (52.7)	47 (55.3)	0.891
		CT	32 (35.2)	27 (31.8)	
		TT	11 (12.1)	11 (12.9)	
	Dominant	T	43 (47.3)	39 (44.7)	0.735
		CC	48 (52.7)	47 (55.3)	
	Recessive	TT	11 (12.1)	11 (12.9)	0.864
		C	80 (87.9)	74 (87.1)	
	Allele	**C**	128 (70.3)	121 (71.2)	0.861
		**T**	54 (29.7)	49 (28.8)	

*SNP* single nucleotide polymorphism. Significant values are in bold.

χ^2^-test.

Table 3 indicates the ORs (95% CI) of patients with COVID-19 hospitalization based on genotypes of different SNPs in vitamin D receptor gene. The genotype of rs11568820 SNP had an inverse association with hospitalization of patients with COVID-19 in crude and after adjustment of comorbidities including coronary heart disease, current smoker and BMI. It was related with 82% reduction in risk of the hospitalization of these patients (OR 0.18, 95% CI: 0.04, 0.88; *P* = 0.034). While, there was no significant relationship between genotypes of SNPs rs7970314 and rs4334089 and hospitalization of patients with COVID-19.

**Table 3:** Odds ratio (95% confidence interval) of patients with COVID-19 hospitalization based on genotypes of different single nucleotide polymorphisms of vitamin D receptors.

SNPs	Models	Genotype	Crude model		Adjusted model*	
			OR (95% CI)	*P* value	OR (95% CI)	*P* value
rs11568820	Genotypic	CT versus CC	1.44 (0.76, 2.72)	0.262	1.76 (0.85, 3.66)	0.129
		TT versus CC	0.20 (0.05, 0.74)	0.016	0.18 (0.04, 0.88)	0.034
	Dominant	T versus CC	1.00 (0.55, 1.08)	0.996	1.14 (0.58, 2.25)	0.686
	Recessive	TT versus C	0.17 (0.05, 0.61)	0.007	0.14 (0.31, 0.67)	0.014
rs7970314	Genotypic	AG versus AA	1.17 (0.61, 2.25)	0.626	1.04 (0.49, 2.21)	0.912
		GG versus AA	0.51 (0.21, 1.25)	0.143	0.63 (0.24, 1.65)	0.346
	Dominant	G versus AA	0.93 (0.51, 1.71)	0.827	0.89 (0.44, 1.78)	0.746
	Recessive	GG versus A	0.47 (0.21, 1.08)	0.076	0.61 (0.25, 1.49)	0.282
rs4334089	Genotypic	CT versus CC	0.86 (0.45, 1.65)	0.654	0.74 (0.34, 1.58)	0.432
		TT versus CC	1.02 (0.40, 2.58)	0.965	0.92 (0.300, 2.82)	0.886
	Dominant	T versus CC	0.90 (0.50, 1.63)	0.735	0.78 (0.39, 1.57)	0.485
	Recessive	TT versus C	1.08 (0.44, 2.64)	0.864	1.03 (0.35, 3.05)	0.947

*SNP* single nucleotide polymorphism, *OR (95% CI)* odds ratio (95% confidence interval).

*Adjusted for comorbidities including coronary heart disease (yes/no), current smoker (yes/no); body mass index (kg/m^2^).

By comparing all applied models in studied SNPs, a significant association with patients’ hospitalization were detected for the rs11568820 in recessive model (OR 0.14, 95% CI: 0.31, 0.67; *P* = 0.014). However, there were no significant relationship in other SNPs.

## Discussion

The present study indicated a potential link between rs11568820 polymorphism and COVID-19 severity. Our results suggested that rs11568820 T variant was associated with a lower risk for severe COVID-19. Based on recessive and codominant genetic models for rs11568820 polymorphism in patients with COVID-19, we found that decreased susceptibility to hospitalization in recessive model but not dominant model.

To date, several studies have examined the association between VDR polymorphisms and COVID-19 severity or susceptibility^8,18–26^. However, findings from earlier studies are controversial. These studies showed a relationship between COVID-19 severity and the VDR polymorphisms of *FokI* (rs2228570, C > T), *TaqI* (rs731236, A > G), *BsmI* (rs1544410, G > A) and *CYP2R1* gene (rs10741657, G > A)^8,18,21,23–25^. However, available evidence providing a link between VDR polymorphisms and COVID-19 severity is not strong and might be false positive^27^. In a similar study, hospitalized patients infected with COVID-19 and suspected COVID-19 patients with mild symptoms were assessed. This study showed a significant association between rs2228570, the CC and the CT genotypes of rs2228570 polymorphisms and susceptibility to COVID-19 infection. However, they could not find any significant differences in the distribution of genotype and allele of VDR between severe and mild patients^23^. It is also possible that inter-individual variations in genomic responses to different levels of vitamin D and epigenetic factors lead to different metabolomic profiles which affect the susceptibility to COVID-19 infection^28,29^. Therefore, it is necessary to examine such associations in different populations.

A growing body of evidence suggests an association between VDR polymorphisms and susceptibility to diverse infectious diseases. The results of a previous study illustrated that rs11568820 SNP was related to shortness of breath in patients with COVID-19^8^. Consistent with these results, we observed significant difference in rs11568820 genotypic distribution between non-hospitalized and hospitalized patients. The rs11568820 has a functional binding site on the VDR gene for the transcription factor Cdx-2. Our results showed that TT genotype was more frequent in non-hospitalized patients, while CT genotype was more prevalent in hospitalized patients. In addition, this association was evident in recessive but not dominant model. Thus, the TT genotype might be a protective factor for severe COVID-19. Cdx-2 is an intestinal-specific transcriptional factor which is not expressed by immune cells; however, it is associated with the risk of some infections such as tuberculosis^30^. The reason behind this association is not fully understood but it is likely that VDR methylation influences the effects of Cdx-2 and confounds the observed relationships between VDR polymorphisms to disease^30^.

Our study has several limitations which should be taken into account when interpreting our results. Our study was a descriptive analysis and cannot reveal any causal relationship between VDR polymorphisms and COVID-19 severity. We also failed to measure serum vitamin D levels due to financial limitations which may independent of VDR SNPs affect the susceptibility to COVID-19 infection and its severity. The lack of data on immunological and inflammatory factors which modulate immune responses did not allow us to control their confounding effects and therefore our results should be interpreted cautiously. Furthermore, due to the small sample size, we failed to explore the associations in various subgroups, such as age and the history of non-communicable disease, which should be considered in future studies. This study is one of the few studies which examined differences in the distribution of VDR SNPs between mild/moderate and severe patients with COVID-19. Earlier studies have mainly focused on the association between serum concentrations of vitamin D and susceptibility to COVID-19 disease.

In conclusion, we found that the TT genotype of rs11568820 was a protective factor for severe COVID-19. However, further studies with a large sample size which consider various confounding factors are warranted to confirm our results.

## Data Availability

The datasets used and/or analyzed during the current study available from the corresponding author on reasonable request. Questionnaires used in the study are prepared by authors.

## References

[1] Richardson S (2020). Presenting characteristics, comorbidities, and outcomes among 5700 patients hospitalized with COVID-19 in the New York city area. JAMA.

[2] Pereira M (2022). Vitamin D deficiency aggravates COVID-19: Systematic review and meta-analysis. Crit. Rev. Food Sci. Nutr..

[3] Hansdottir S (2011). Vitamin D effects on lung immunity and respiratory diseases. Vitam. Horm..

[4] Grant WB (2020). Evidence that vitamin D supplementation could reduce risk of influenza and COVID-19 infections and deaths. Nutrients.

[5] Ebadi M, Montano-Loza AJ (2020). Perspective: Improving vitamin D status in the management of COVID-19. Eur. J. Clin. Nutr..

[6] Bilezikian JP (2020). Mechanisms in endocrinology: Vitamin D and COVID-19. Eur. J. Endocrinol..

[7] Bizzaro G (2017). Vitamin D and autoimmune diseases: Is Vitamin D receptor (VDR) polymorphism the culprit?. Isr. Med. Assoc. J..

[8] Abdollahzadeh R (2021). Association of Vitamin D receptor gene polymorphisms and clinical/severe outcomes of COVID-19 patients. Infect. Genet. Evol..

[9] Jolliffe DA (2018). Vitamin D receptor genotype influences risk of upper respiratory infection. Br. J. Nutr..

[10] Li Z (2020). Advances in single nucleotide polymorphisms of Vitamin D metabolic pathway genes and respiratory diseases. J. Adv. Med. Sci..

[11] Sarrafzadegan N (2022). Isfahan COVID cohort study: Rationale, methodology, and initial results. J. Res. Med. Sci..

[12] Haghighatdoost F (2023). Coffee consumption and risk of hypertension in adults: Systematic review and meta-analysis. Nutrients.

[13] Palipudi KM (2016). Methodology of the global adult tobacco survey—2008–2010. Glob Health Promot.

[14] Geramian N (2014). Development of a questionnaire to assess drug abuse among high school students of Isfahan province, Iran: An action research. Int. J. Prev. Med..

[15] Craig CL (2003). International physical activity questionnaire: 12-country reliability and validity. Med. Sci. Sports Exerc..

[16] World Health Organization. *WHO STEPS Surveillance: Guide to Physical Measurements* (2008).

[17] Miller SA, Dykes DD, Polesky HF (1988). A simple salting out procedure for extracting DNA from human nucleated cells. Nucleic Acids Res.

[18] Apaydin T (2022). Effects of Vitamin D receptor gene polymorphisms on the prognosis of COVID-19. Clin. Endocrinol..

[19] Freitas AT (2021). Vitamin D-related polymorphisms and vitamin D levels as risk biomarkers of COVID-19 disease severity. Sci. Rep..

[20] Al-Anouti, F., et al., *Associations between Genetic Variants in the Vitamin D Metabolism Pathway and Severity of COVID-19 among UAE Residents.* Nutrients, 2021. **13**(11). 10.3390/nu13113680 PMC8625365 34835935

[21] Balzanelli MG (2022). Analysis of gene single nucleotide polymorphisms in COVID-19 disease highlighting the susceptibility and the severity towards the infection. Diagnostics (Basel).

[22] Kotur N (2021). Association of Vitamin D, zinc and selenium related genetic variants with COVID-19 disease severity. Front. Nutr..

[23] Jafarpoor A (2022). VDR gene polymorphisms are associated with the increased susceptibility to COVID-19 among iranian population: A case-control study. Int. J. Immunogenet..

[24] Albu-Mohammed WHM, Anvari E, Fateh A (2022). Evaluating the role of BglI rs739837 and TaqI rs731236 polymorphisms in Vitamin D receptor with SARS-CoV-2 variants mortality rate. Genes (Basel).

[25] Mamurova, B. *et al*. *A Strong Association Between the VDR Gene Markers and SARS-CoV-2 variants* (2022).

[26] Peralta EM (2021). TaqI polymorphism of the VDR gene: Aspects related to the clinical behavior of COVID-19 in Cuban patients. Egypt. J. Med. Hum. Genet..

[27] Charoenngam N (2023). Genetic variations of the Vitamin D metabolic pathway and COVID-19 susceptibility and severity: Current understanding and existing evidence. Biomedicines.

[28] Shirvani A (2019). Disassociation of Vitamin D’s calcemic activity and non-calcemic genomic activity and individual responsiveness: A randomized controlled double-blind clinical trial. Sci. Rep..

[29] Carlberg C (2013). Primary Vitamin D target genes allow a categorization of possible benefits of Vitamin D_3_ supplementation. PLoS One.

[30] Meyer V, Bornman L (2018). Cdx-2 polymorphism in the vitamin D receptor gene (VDR) marks VDR expression in monocyte/macrophages through VDR promoter methylation. Immunogenetics.

